# Machine learning based classification of cells into chronological stages using single-cell transcriptomics

**DOI:** 10.1038/s41598-018-35218-5

**Published:** 2018-11-21

**Authors:** Sumeet Pal Singh, Sharan Janjuha, Samata Chaudhuri, Susanne Reinhardt, Annekathrin Kränkel, Sevina Dietz, Anne Eugster, Halil Bilgin, Selçuk Korkmaz, Gökmen Zararsız, Nikolay Ninov, John E. Reid

**Affiliations:** 1 Center for Molecular and Cellular Bioengineering, TU Dresden, Dresden, 01307 Germany; 2 Paul Langerhans Institute Dresden of the Helmholtz Center Munich at the University Hospital Carl Gustav Carus of TU Dresden, Dresden, 01307 Germany; 3 Max Planck Institute of Molecular Cell Biology and Genetics, Dresden, 01307 Germany; 4 B CUBE-Center for Molecular Bioengineering, TU Dresden, Dresden, 01307 Germany; 5 Department of Computer Engineering, Abdullah Gül University, Kayseri, 38030 Turkey; 6 Department of Biostatistics and Medical Informatic, Trakya University, Edirne, 22030 Turkey; 7 Department of Biostatistics, Erciyes University, Kayseri, 38030 Turkey; 8 Turcosa Analytics Solutions Ltd. Co., Erciyes Teknopark 5, Kayseri, 38039 Turkey; 9 MRC Biostatistics Unit, University of Cambridge, Cambridge, CB2 0SR UK; 10 The Alan Turing Institute, London, NW1 2DB UK

## Abstract

Age-associated deterioration of cellular physiology leads to pathological conditions. The ability to detect premature aging could provide a window for preventive therapies against age-related diseases. However, the techniques for determining cellular age are limited, as they rely on a limited set of histological markers and lack predictive power. Here, we implement GERAS (GEnetic Reference for Age of Single-cell), a machine learning based framework capable of assigning individual cells to chronological stages based on their transcriptomes. GERAS displays greater than 90% accuracy in classifying the chronological stage of zebrafish and human pancreatic cells. The framework demonstrates robustness against biological and technical noise, as evaluated by its performance on independent samplings of single-cells. Additionally, GERAS determines the impact of differences in calorie intake and BMI on the aging of zebrafish and human pancreatic cells, respectively. We further harness the classification ability of GERAS to identify molecular factors that are potentially associated with the aging of beta-cells. We show that one of these factors, *junba*, is necessary to maintain the proliferative state of juvenile beta-cells. Our results showcase the applicability of a machine learning framework to classify the chronological stage of heterogeneous cell populations, while enabling detection of candidate genes associated with aging.

## Introduction

Aging is a universal phenomenon, during which cells undergo progressive transcriptional^1,2^, genomic^3,4^, epigenetic^5^, and metabolic^6^ changes. The age-related modifications can deteriorate the functional properties of cells. The accumulation of cellular defects can lead to a decline in organismal health and to the onset of age-related diseases. A major focus of the biology of aging is to identify factors that accelerate or slow-down the cellular aging process. Biological studies have identified multiple modifiers of the aging process, including genetic and environmental factors^7,8^. For instance, caloric restriction has been demonstrated to increase lifespan in multiple species^9^, including humans^10^. However, the discovery of factors that influence aging relies on retrospective analysis, after the impact of age has already manifested itself, and depends on a restricted set of indicators based on histological analysis^11^. It is therefore imperative to develop reliable indicators of cellular age that forgo the need for detrimental phenotypes. Predicting cellular aging before the defects manifest themselves would provide a window for therapeutic interventions. Preventive therapies during this window would bypass additional complications arising after the onset of the pathology.

The development of a reliable cellular age classifier requires at least two factors. Firstly, it entails a reliable assessment of the progressive changes cells undergo with age. Secondly, the classifier should be capable of placing cells of unknown age along a transition path in order to estimate their age. Recent advances in single-cell mRNA expression profiling have enabled assessment of the transcriptional changes cells undergo with age^12^. Cellular progression through the transcriptional transitions is increasingly being described by both heuristic methods and probabilistic models. These methods are categorized as pseudotemporal estimation algorithms and use techniques such as dimensionality reduction, graph theory, bifurcation analysis and optimal-transport analysis to place cells along a transition trajectory^13–18^. Although current methods can reveal cellular transitions during a differentiation process^19–22^, they have only been shown to work retrospectively, that is they have limited ability to insert *de-novo* samples into the trajectories.

Positioning of *de-novo* samples in a cellular aging trajectory requires discrimination of the transcriptional features of importance from the confounding factors that accompany single-cell transcriptome measurements. The three main confounding factors are: (1) biological noise due to fluctuations in mRNA expression levels, (2) technical noise inherent in single-cell mRNA sequencing, and (3) cell-type diversity within an organ. Biological noise can arise due to the stochasticity in biochemical processes involved in mRNA production and degradation^23,24^, heterogeneity in the cellular microenvironment^25^, and many more unknown factors. Technical noise, on the other hand, arises due to the sensitivity and depth of single-cell sequencing technology^26^. Sequencing involves conversion of mRNA into cDNA and amplification of the minute amounts of cDNA. These steps could omit certain mRNA molecules, muting their detection. Moreover, amplified cDNA molecules might escape sequencing due to the limits on the comprehensiveness of the technology. In effect, expression noise is inherent to single-cell measurements of mRNA expression levels.

The diversity in cell types within an organ adds an additional layer of complexity to the inherent noise in mRNA expression. Moreover, numerous studies have demonstrated the presence of cellular sub-populations even among nominally homogenous cells^27,28^. For example, pancreatic beta-cells have been shown to consist of dynamic sub-populations with different proliferative and functional properties^29–31^, and liver cells were demonstrated to display variability in gene expression depending on their location within the organ^32^. Thus, the inherent cell-to-cell heterogeneity adds to the challenge of extracting age-related transcriptional changes from mRNA expression profiles. Furthermore, cellular heterogeneity makes it difficult to extrapolate the results from studies at the tissue-scale to the aging of individual cells and to identify common molecular signatures of aging^33,34^.

In this study, we provide a framework that efficiently ‘learns’ the cellular transitions of aging from single-cell gene expression data in the presence of expression noise and cellular heterogeneity. Our age classifier is trained to recognize the age of individual cells based on their chronological stage. Chronological stage is easy to define, and hence provides a ground truth for the training. To show the utility of the stage classifier, we apply it to the pancreatic beta-cells, which represent an excellent system for studying aging. In mammals, the beta-cell mass is established during infancy and serves the individual throughout life^35^. The long-lived beta-cells support blood glucose regulation, with their dysfunction implicated in the development of Type 2 diabetes. Older beta-cells display hallmarks of aging, such as a reduced proliferative capacity^36^ and impaired function^37^. We first focus on the zebrafish beta-cells due to the potential for visualization and genetic manipulation at single-cell resolution^31,36^, and extend our framework to human pancreatic cells using publicly available published datasets. Finally, we demonstrate the classifier’s utility in identifying the impact of environmental factors on aging.

## Results

### Machine learning based framework accurately and robustly classifies chronological stage

To capture the transcriptional dynamics of beta-cells with age, we performed single-cell mRNA sequencing of beta-cells in primary islets dissected from animals belonging to seven ages of zebrafish: 1 month post-fertilization (mpf), 3 mpf, 4 mpf, 6 mpf, 10 mpf, 12 mpf and 14 mpf. For classification, the seven ages were divided into three chronological stages: ‘Juvenile’ (1 mpf), ‘Adolescent’ (3, 4 and 6 mpf) and ‘Adult’ (10, 12 and 14 mpf). Using *Tg(ins:Betabow)*^31^, a transgenic line that specifically marks zebrafish beta-cells with red fluorescence (Supplementary Fig. S1), we isolated and sequenced 827 beta-cells in four independent batches (Supplementary Table S1). Sequencing was performed using the Smart-Seq2 protocol, which has been demonstrated to provide higher transcriptional coverage than other methods^38^. The sequenced cells were quality-controlled to yield a total of 637 beta-cells (Supplementary Fig. S2). We tested statistical models for classifying the cells using the ground truth provided by the chronological stage of the cellular origin: ‘Juvenile’, ‘Adolescent’ or ‘Adult’. As input to the classifiers, genes were ranked in descending order of their variability (calculated using the median absolute deviation for gene expression) and the top variable genes were selected for training (Supplementary Table S2). We first tested a multinomial logistic regression classifier using the ‘nnet’^39^ package in R. For training the regression model, 80% of the beta-cells were randomly chosen. Using the trained model, we evaluated the classification accuracy on the test set comprising the remaining 20% of the cells. The cells of the test set had not been used to train the model. The multinomial logistic regression model displayed an overall accuracy (proportion of cells for which the classification stage matched the actual stage) of 64% (Supplementary Fig. S3).

**Figure 1:**
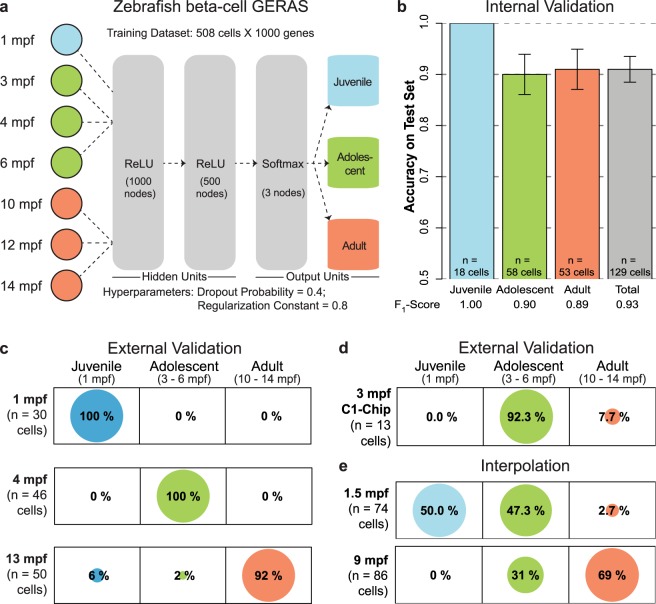
A Chronological age classifier for zebrafish beta-cells. (**a**) Schematic of the machine learning framework for classifying the chronological stage of zebrafish beta-cells based on single-cell transcriptome (see Online Methods for details). (**b**) Barplot showing the accuracy of GERAS for classifying the ages of beta-cells that were excluded during the training of the model. The classification of the excluded beta-cells displayed greater than 91% accuracy. Error bars indicate standard error. The F_1_-score for each stage is displayed at the bottom. The F_1_-score is a metric evaluating the precision and the sensitivity of the classifier, with the highest being 1, and the lowest being 0. (**c**) Balloonplots showing the age-classification of *de-novo* sequenced beta-cells. GERAS classified the age of the cells from independent sources with greater than or equal to 92% accuracy, showcasing the robustness of the model in handling biological and technical noise. (**d**) Balloonplots showing the age-classification of beta-cells from 3 mpf animals sequenced using the Fluidigm C1 platform. GERAS classified the age of the cells from the cohort with 92.3% accuracy, demonstrating the robustness of the model in handling alternative sequencing pipelines. (**e**) The capacity of GERAS to perform interpolation was tested using cells with ages in-between the chronological stages used to train GERAS. More than 97% of the cells from the intermediate time-points classify in the nearest-neighbor stages. Number of cells for each condition is denoted by ‘n’.

We next developed a supervised deep learning classifier based on neural networks to perform the classification task (Fig. 1a). Since neural networks are prone to overfitting, two normalizing hyperparameters were added: L2 regularization (which penalizes a strong focus on few inputs) and dropout regularization (which helps ‘averaging’ across connections). This framework was named GERAS (GEnetic Reference for Age of Single-cell) in reference to the Greek God of old age. Similar to the logistic regression training, for developing GERAS we utilized 80% of the beta-cells, which were randomly chosen. The input to GERAS consisted of 1000 genes with the highest gene expression variability estimated using the median absolute deviation (Supplementary Table S2). The optimal normalizing hyperparameters (L2 and dropout regularization) were determined by cross-validation and used for training the final classifier. Using the trained GERAS, internal validation was carried out with a test set comprising the remaining 20% of the beta-cells. The cells of the test set had not been used to train GERAS. This internal validation achieved an overall accuracy (proportion of cells for which the classification stage matched the real stage) of 91 ± 3% (Fig. 1b). This suggests that the neural-net based GERAS is more accurate than multinomial logistic regression in classifying individual cells into chronological stages. Additionally, the high accuracy (91%) achieved by GERAS shows that cells can be classified into chronological stages based solely on their mRNA expression profile.

For the trained neural network, we estimated the contribution of the 1000 input genes towards classification (Supplementary Fig. S4, Supplementary Table S2). The estimation ranked the input genes in descending order of their importance towards the classification of cells. The importance of each input gene is calculated using the strength (weights) of the neural network connections^40^. Input genes with stronger neural network connections will be more important to the classification task than input genes with weaker connections. The estimation showed that the input genes displayed a wide distribution of importance towards the accuracy of classification. For instance, the gene *ucp2* (importance = 0.53) displayed a stronger importance towards the performance of GERAS as compared to the gene *vegfaa* (importance = 7 ∗ 10^−7^). In this case, the gene *vegfaa* plays an insignificant role towards the classification of beta-cells into chronological stages. Notably, several of the top 20 most important genes were previously implicated in diabetes (Supplementary Fig. S4b).

We wanted to understand the robustness of GERAS under biological and technical noise, typically encountered upon measuring the transcriptome of single-cells in different batches. To this end, we performed external validation using independently sequenced beta-cells. We sequenced new batches of beta-cells from 1 mpf (‘Juvenile’), 4 mpf (‘Adolescent’) and 13 mpf (‘Adult’) animals and used GERAS to classify their chronological stage (Fig. 1c). All cells from the independent cohort of 1 mpf were classified as ‘Juvenile’ (100% accuracy), the ground truth for the stage of the cells. Similarly, the 4 mpf cohort displayed 100% accuracy. Lastly, within the 13 mpf independent cohort, 46 out of 50 beta-cells were classified in the ‘Adult’ stage, thus demonstrating a 92% accuracy in determining the ground truth of the sample (Fig. 1c). Additionally, we tested the performance of GERAS with beta-cells sequenced using alternative pipelines. Specifically, we utilized the C1-Chip platform from Fluidigm to sequence a new batch of beta-cells from adolescent animals (3 mpf). GERAS achieved 92.3% success in correctly classifying the cells from the new batch as ‘Adolescent’ (Fig. 1d). These data underscore the potential of GERAS in effectively handling batch effects.

To investigate the performance of GERAS on interpolation tasks, we assessed the model’s ability to classify cells obtained from time-points in-between the discrete chronological stages we used for training. Thus, we collected beta-cells from animals aged 1.5 and 9 mpf, which fall in-between the ‘Juvenile/Adolescent’ or the ‘Adolescent/Adult’ stages, respectively. GERAS classified 50% of the beta-cells from 1.5 mpf animals as ‘Juvenile’, and 47.3% as ‘Adolescent’ (Fig. 1e). For the new set of 9 mpf beta-cells, 31% of the cells were classified as ‘Adolescent’, and 69% as ‘Adult’ (Fig. 1e). None (0%) of the cells were attributed to the ‘Juvenile’ stage. Overall, GERAS classified 97.3% of the 1.5 mpf beta-cells and 100% of the 9 mpf beta-cells in stages neighboring the actual age of the sample. Additionally, in our framework, chronological stage determination can be easily converted to classification probability by using the output of ‘softmax’ layer (Fig. 1a and Methods). This transforms discrete classifications into a continuous probability distribution. The ‘softmax’ layer provides the probability (0 being lowest, and 1 being highest) for a cell to be classified into each of the three chronological stages: ‘Juvenile, ‘Adolescent’, and ‘Adult’. Beta-cells from 1.5 mpf animals displayed statistically similar probability to be classified into the ‘Juvenile’ or ‘Adolescent’ stage (Supplementary Fig. S5a) (p-value > 0.05, Tukey’s test) and lower probability to be classified into the more distant ‘Adult’ stage (p-value < 0.001, Tukey’s test). Thus, GERAS is equally likely to classify the 1.5 mpf cells to ‘Juvenile’ or ‘Adolescent’, but not ‘Adult’ stages, which lays further apart in time. Similarly, the classification probability for beta-cells from 9 mpf animals displayed a trend towards classification in the ‘Adult’ stage over ‘Adolescent’ stage, with close to zero probability for classification into the ‘Juvenile’ stage (Supplementary Fig. S5b). It is important to note that the 9 mpf time-point lies closer to the ‘Adult’ (10–14 mpf) stage as comparted to the ‘Adolescent’ (3–6 mpf), which possibly shifts the classification probability closer to the ‘Adult’ stage. However, the low probability for classification into the chronologically distant ‘Juvenile’ stage (mean = 0.03) strengthens the interpolation capacity of GERAS. Overall, our results demonstrate that our model can efficiently handle samples obtained from time-points in-between the discrete chronological stages.

### GERAS evaluates the impact of environmental and molecular factors on cellular age

**Figure 2:**
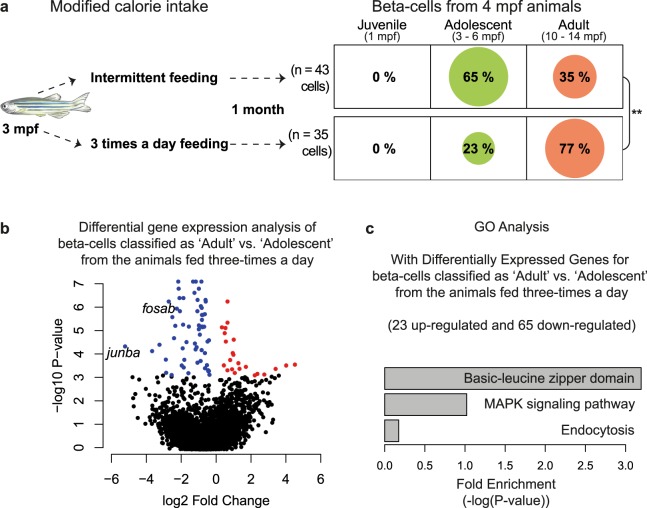
Impact of calorie intake on the chronological stage of zebrafish beta-cells. (**a**) The impact of calorie intake on the classification stage of beta-cells was investigated. A higher proportion of beta-cells from the animals fed three-times-a-day were classified by GERAS as ‘Adult’, as compared to cells from animals on intermittent feeding, in which a majority of the cells (65%) were classified as adolescent. (Fisher’s Exact Test, **p-value < 0.01). (**b**) Volcano plot summarizing the differential expression analysis of beta-cells classified as ‘Adult’ or ‘Adolescent’ from the animals fed three-times a day. Colored dots represent significantly regulated genes (false-discovery rate (fdr) < 0.05). Red dots represent up-regulated genes, while blue dots represent down-regulated genes. (**c**) Gene-ontology (GO) analysis of the differentially regulated genes using DAVID^42^. This analysis includes the down-regulated (blue in b) and up-regulated (red in b) genes. Zebrafish illustration provided with permission from Priyanka Oberoi.

The rate of aging is susceptible to modifications^8^ and nutritional cues have been noted to alter aging in many organisms^9,10^. To investigate the effect of altering nutritional cues on cellular age, we employed the ability of GERAS to handle batch effects and interpolation. Specifically, we focused on studying the impact of calorie intake on beta-cell aging. We separated 3 mpf adolescent zebrafish siblings into two groups. One group was fed three times a day with *Artemia*, a typical fish diet consisting of living prey with a relatively high amount of fat and carbohydrates^41^. The other group was placed on intermittent feeding with normal feeding performed on alternate days (Fig. 2a). After one month, the beta-cells were isolated and GERAS was applied to classify beta-cell age in each group. The analysis showed a striking difference in the age-classification of the two sets of beta-cells (Fig. 2a). While 65% of the beta-cells from zebrafish on intermittent feeding were classified as ‘Adolescent’, only 23% of the beta-cells from three-times-a-day-fed animals were similarly classified; the rest 77% were categorized as ‘Adult’. This difference in classification suggests that higher-caloric intake expedites the aging of beta-cells.

We performed differential gene-expression analysis of beta-cells that were classified by GERAS as either ‘Adult’ or ‘Adolescent’ (Fig. 2b, Supplementary Table S3) in the three-times-a-day-fed group. These analysis identified enrichment of 23 and 65 genes in beta-cells classified as ‘Adolescent’ and ‘Adult’, respectively. Unbiased gene ontology analysis using DAVID^42^ revealed involvement of the differentially regulated genes in pathways related to MAPK signaling, endocytosis, and basic-leucine zipper domain (bZIP) containing transcription factors (Fig. 2c). Specifically, the comparison suggests that the ‘Adult’ beta-cells exhibit down-regulation of bZIP-domain containing transcription factors *junba* and *fosab*. Notably, both *junba* and *fosab* also displayed significant down-regulation with age in our primary mRNA expression data of beta-cells from three chronological stages, (t-test using the ROTS package^43^, p < 0.001) (Supplementary Fig. S6). However, when comparing the transcriptome of the beta-cells isolated from three-times-a-day fed animals (n = 35 cells) to the beta-cells isolated from animals on intermittent feeding (n = 43 cells), *junba* is not identified to be significantly downregulated (Supplementary Table S4). Thus, GERAS-based classification of beta-cells into ‘Adolescent’ and ‘Adult’ sub-populations was necessary to identify potential genes that change in expression with aging at the single-cell level.

**Figure 3:**
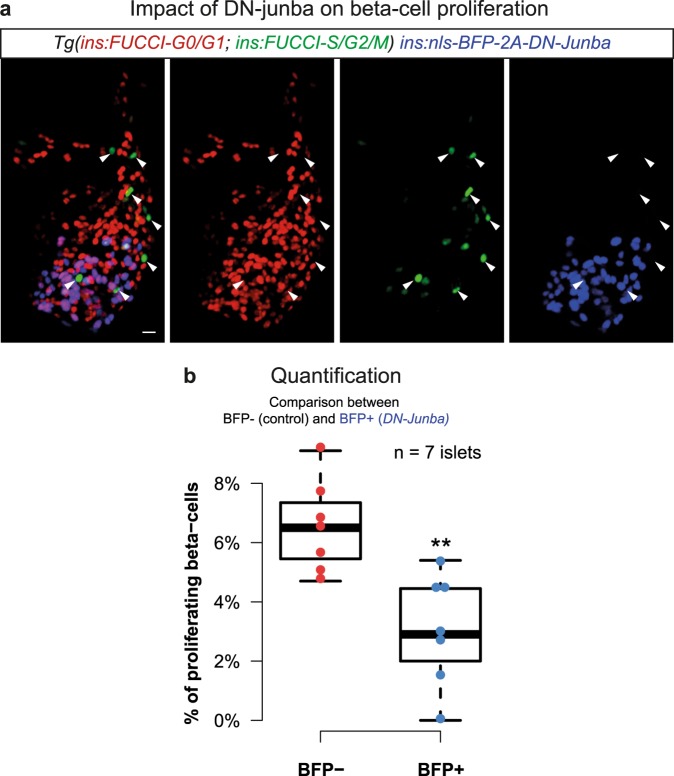
Inhibition of *junba* reduces the proliferation of zebrafish beta-cells. (**a**) Maximum intensity confocal projections of beta-cells from a 30 dpf animal showing mosaic expression of *nls-BFP-2A-DN-junba* (blue) together with *Tg(ins:FUCCI-S/G2/M)* (green) and *Tg(ins:FUCCI-G0/G1)* (red). Arrowheads mark proliferating beta-cells, as indicated by the presence of green fluorescence and absence of red fluorescence. Scale bar 10 μm. (**b**) Tukey-style boxplots showing the percentage of proliferating beta-cells among BFP+ and BFP- cells. BFP+ cells co-express *DN-junba*, while the BFP- cells act as internal control. The BFP+ cells show a significant decrease in the proportion of proliferating cells (t-test, **p-value < 0.01). ‘n’ denotes number of islets.

To investigate the biological impact of reducing *junba* function in beta-cells, we overexpressed a dominant negative version of *junba* specifically in beta-cells (using an *ins:nls-BFP-2A-DN-junba* construct) (Supplementary Fig. S7a). The expression of *nls-BFP-2A-DN-junba* was induced in the background of the beta-cell specific fluorescence ubiquitination cell cycle indicator (FUCCI)-reporters^44,45^, allowing identification of beta-cell’s cell-cycle stage (Supplementary Fig. S7b,c). Comparison between the juxtaposed *DN-junba*-expressing and control cells within islets from juveniles (1 mpf), a stage associated with high rates of beta-cell proliferation^44^, showed a 50% decline in proliferation upon *DN-junba* expression (Fig. 3a,b). Thus, blocking *junba* function can reduce the proliferation of juvenile beta-cells. Since the reduction in proliferation of beta-cells is a hallmark of aging^36,37]^, our results suggest that declining *junba* expression might underlie this reduction.

### A single model for chronological stage classification of the entire human pancreatic cells

**Figure 4:**
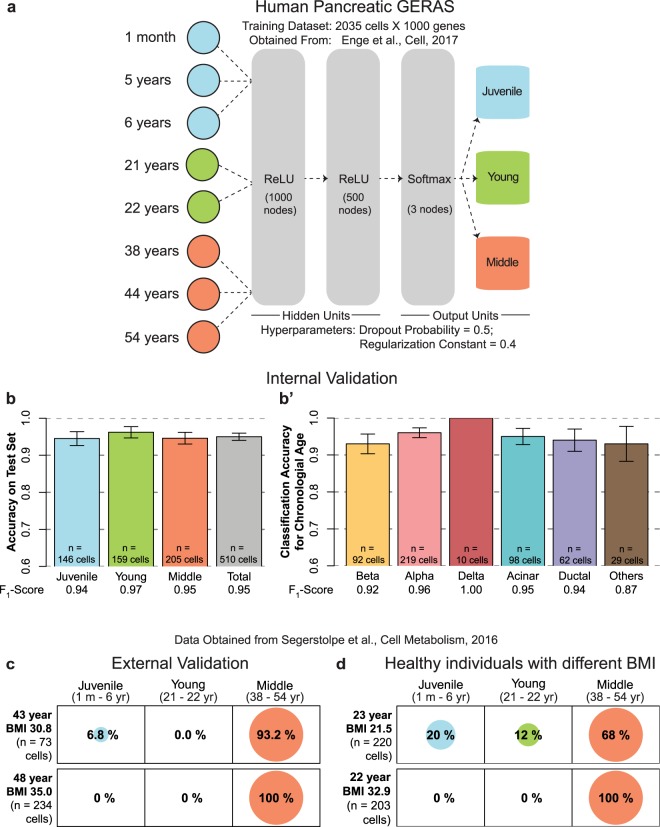
A Chronological age classifier for human pancreatic cells. (**a**) A single chronological age classifier for the entire ensemble of human pancreatic cells using machine learning. No cell-type segregation was performed during training. (**b**) Barplot showing the accuracy of GERAS on classifying the age of pancreatic cells that were not used for training the model. An accuracy of 95% was achieved for cells previously unseen by GERAS. (b’) The classification accuracy of GERAS on the previously unseen pancreatic cells after segregating them into major cell-types. Classification accuracy equals the proportion of cells for which the classification stage matched the actual stage. For each cell-type, greater than 93% accuracy was achieved. Error bars indicate standard error. F_1_-scores, a measure of precision and sensitivity of the classifier, are depicted at the bottom. (**c**) External validation for the classifier was provided by human pancreatic single-cell mRNA expression data obtained from an independent publication. Cells from individuals belonging to the ‘Middle’ (38–54 years) stage of the classifier displayed greater than 93% accuracy. (**d**) Balloonplot showing classification of cells from individuals with similar chronological age but different BMI. In individuals with normal BMI, 32% of the cells were classified in ‘Juvenile’ and ‘Young’ stages, while none (0%) of the cells from individuals with obese BMI were similarly classified. Number of cells for each condition is denoted by ‘n’.

Next, to test the applicability of our framework beyond the scope of zebrafish beta-cells, we developed a classifier for human cells using the entire ensemble of pancreatic cells. The pancreas, a gland located in the abdomen, is involved in metabolic regulation and food digestion. Metabolic regulation is accomplished by the endocrine part of the pancreas, which chiefly consists of beta-, alpha-, and delta-cells. Food digestion, on the other hand, is contributed by the exocrine part of the pancreas, composed of ductal and acinar cells. An important characteristic of pancreatic cells is the presence of cell-specific marker genes, allowing computational segregation of the various cell-types based on mRNA expression levels (Methods). To develop the classifier for human pancreatic cells, we obtained single-cell mRNA expression profiles from Enge *et al*. Their study generated single-cell transcriptomes from pancreatic cells of eight healthy individuals belonging to three discrete stages^46^: ‘Juvenile’ (1 month, 5 and 6 years), ‘Young’ (21 and 22 years), and ‘Middle’ (38, 44 and 54 years) (Fig. 4a). Without segregating the data by cell-type, we trained GERAS to classify the chronological stage for the entire ensemble of pancreatic cells. The trained GERAS, utilizing inputs from multiple genes (Supplementary Fig. S8, Supplementary Table S5), achieved an overall accuracy of 95% on the test set (Fig. 4b). Upon segregating the results by cell type, based on the expression of their respective markers, we found that GERAS displayed >90% accuracy for each major cell-type of the pancreas (Fig. 4b’), demonstrating the feasibility of developing a single age classifier for the multiple cell types of the pancreas.

As an additional validation, a second assessment with human cells was undertaken by utilizing the single-cell mRNA expression profiles of human pancreatic cells from a publication by Segerstolpe *et al*.^47^. This independent cohort contains single-cell transcriptomes from pancreata of six healthy individuals ranging from 22–48 year of age. Additionally, the body mass index (BMI) for each individual was reported, allowing comparisons between individuals with similar chronological age but different body weight. Using GERAS trained with the human data from Enge *et al*., we classified the chronological stage of the cells from two individuals (aged 43 and 48 years) belonging to the ‘Middle’ age group (38–54 years). The classifications displayed >93% accuracy (Fig. 4c). This high classification accuracy on data from a second independent source further strengthens the external validation of our model. Next, we utilized the data from two individuals, aged 23 and 22 years. Despite the proximity in their chronological age, these two individuals differed in their BMI values (21.5 – normal and 32.9 – obese, respectively). Strikingly, while 32% of the cells from the 23 year old with normal BMI were classified in the ‘Juvenile’ or ‘Young’ stage, none of the cells from the 22 year old with obese BMI fell in the two stages (Fig. 4d). Following this observation, we calculated the classification probability of the all six individuals in relation to their BMI. The probability results from our analysis suggest that an obese BMI correlates with an increased probability for the cells to be classified in an older stage (Supplementary Fig. S9). We recommend exercising caution while interpreting this result due to the multiple confounding factors associated with human samples that we could not control for. A GERAS developed with cells from individuals encompassing a wider distribution of age and BMI range would be desired for stronger conclusions. Nevertheless, the successful age classification of an entire human organ and its external validation, demonstrate the adaptability of our framework to diverse cell-types, thereby establishing the universality of the approach.

## Discussion

In this study, we have presented a method that provides the blueprint for developing a classifier for cellular aging. Our chronological stage classifier efficiently handles biological and technical noise, and functions robustly on a diverse cell population. The temporal classifier was developed in an unbiased, data-driven manner. Genes for building the classifier were not selected based on their differential expression with time. The classifier predicted the chronological stage solely from the expression profile of the top 1000 most variable genes. The algorithm, however, did not use all genes uniformly. Instead, varying levels of importance were attributed to the input genes (Supplementary Fig. S4 and S8). Multiple genes exhibiting high importance for classification show an existing association with metabolic and age-related degenerative disorders. For instance, the human pancreatic GERAS ascribes high importance to Amyloid precursor protein (APP) (Supplementary Fig. S8b), which is associated with Alzheimer’s disease, and also recently implicated in pancreatic biology^48^. In the future, it would be worthwhile to test the biological functions for the genes selected by the classifier, and to follow-up on them as potential biomarkers of the aging process.

We utilized the framework to determine the impact of calorie intake on the aging of zebrafish beta-cells. Specifically, a higher percentage of beta-cells isolated from animals fed three-times-a-day were classified in the ‘Adult’ stage as compared to beta-cells isolated from animals on intermittent feeding (Fig. 2a). Differential gene-expression analysis of beta-cells from the three-times-a-day group, which were classified as ‘Adolescent’ or ‘Adult’ showed reduced *junba* expression levels in the latter population (Fig. 2b,c). It is important to note that the classification of beta-cells by GERAS did not utilize *junba* as one of the 1000-input genes (Supplementary Table S2), thereby showing the potential of our approach to identify aging associated factors in an unbiased manner. Follow-up analysis using a genetic technique (Supplementary Fig. S7) verified the role of one candidate gene, *junba*, in regulating the proliferation of beta-cells (Fig. 3). However, a reduction in proliferation represents one aspect of the aging process, and additional roles for *junba* activity during the aging process still need verification. Nonetheless, the age-dependent reduction of *Junb*, the mammalian homologue of *junba*, has been implicated in post-natal maturation of mouse beta-cells^49^. It would be of interest to follow-up on these results and study the connection between aging and *Junb* activity in mammalian models.

Importantly, differential gene expression analysis comparing beta-cells collected from animals fed three-times-a-day to beta-cells collected from animals on intermittent feeding failed to identify *junba* as a molecular factor associated with aging (Supplementary Table S4), which might reflect the inherent heterogeneity of the aging process. The heterogeneity in the age of beta-cells was also observed during the interpolation analysis (Fig. 1d), in which cells from intermediate time-points classified in the two adjacent stages. Asynchronous cellular aging in beta-cells was recently proposed based on histological and molecular markers^36,50^. Quantifying the extent of heterogeneity in the aging process while capturing the mRNA expression profile, made possible by our framework, provides an exciting opportunity for understanding the molecular underpinnings of heterogeneous cellular aging. Importantly, such analysis was possible due to the single-cell-centric nature of our approach, and would be missed out with bulk sequencing in which the cellular variability is averaged out.

Our machine-learning based framework has high flexibility in its design and execution, which can be exploited to develop predictive models based on diverse biological parameters. The inputs to the classifier are not limited to mRNA expression levels but can be extended to include other covariates. With improvements in single cell epigenetics^51^, new models integrating both genetic and epigenetic changes could be built to improve accuracy and resolution.

The classifiers presented in this study are restricted by sequencing platforms and the specific tissues utilized for training them. This limits their immediate adaptation. The classifiers are built with data generated from Smart-Seq2 sequencing pipeline, which captures the full-length mRNAs with high transcriptome coverage. The classifier might be unable to handle the data from Drop-seq or MARS-seq, protocols that sequence the 3′-end of mRNA and provide lower-coverage^38^. Computational efforts for eliminating the idiosyncrasies of individual platforms^52^ would help to remove this restriction. Additionally, the classifiers do not extend beyond the currently described tissues. Investigators interested in the aging of other cells, for instance muscle, would need to develop and validate *de-novo* statistical models. Nevertheless, we expect the groundwork presented here to help with the development of machine learning models. Further improvements of our approach could expedite the identification of age-modifying factors, which are important regulators of development and disease.

## Conclusion

Here we developed a machine learning based platform that assigns individual cells to chronological stages based on the cell’s transcriptome. We show the framework’s robustness in handling multiple sample processing pipelines, time-points that fall between the discrete chronological stages, and diversity in cell types. The framework’s capability to characterize aging factors was demonstrated through evaluation of the impact of a higher-calorie feeding on beta-cell aging. The classification capacity of the framework was further harnessed to discover *junba* as a candidate gene that maintains the proliferative beta-cell state, a characteristic trait of younger beta-cells. Broad applicability of the framework was demonstrated by classifications on the entire human pancreatic tissue. We anticipate that the robustness and flexibility exhibited here will enable the development of aging models for multiple tissues, opening the possibility of detecting premature aging and preventing pathological developments. To maximize the accessibility and impact of the study, the framework is openly shared on github^53^, and a user-friendly, graphical interface is provided for generating classifications from trained models.

## Methods

### Zebrafish strains and husbandry

Wild-type or transgenic zebrafish of the outbred AB, WIK or a hybrid WIK/AB strain were used in all experiments. Zebrafish were raised under standard conditions at 28 °C. Animals were chosen at random for all experiments. Published transgenic strains used in this study were *Tg(ins:BB1.0L; cryaa:RFP)*^31^; *Tg(ins:FUCCI-G1)*^s948 ^^44^; *Tg(ins:FUCCI-S/G2/M)*^s946 ^^44^. Experiments were conducted in accordance with the Animal Welfare Act and with permission of the Landesdirektion Sachsen, Germany (permits AZ 24-9168.11-1/2013-14, TV38/2015, T12/2016, and T13/2017).

### Development of GERAS for zebrafish beta-cells

For development of GERAS for zebrafish beta-cells, read counts were used from seven ages of zebrafish: 1 mpf (n = 87 cells), 3 mpf (n = 109 cells), 4 mpf (n = 88 cells), 6 mpf (n = 90 cells), 10 mpf (n = 92 cells), 12 mpf (n = 111 cells) and 14 mpf (n = 60 cells). The 3 mpf and 6 mpf stages contained two batches of beta-cells collected and sequenced on different days. Each batch of cells originated from six zebrafish. Read counts were normalized to transcripts per million (TPM) using the formula:

$$Transcrip{t}_{gc}=\frac{Read\,Coun{t}_{gc}}{Length\,in\,k{b}_{g}}$$

$$TP{M}_{gc}=\frac{Transcrip{t}_{gc}}{{\sum }_{g}Transcrip{t}_{c}}\ast 1,\,000,\,000$$

where for gene *g* and cell *c*, *Transcript*_*gc*_ are the number of transcripts calculated by dividing the read counts to the length of the gene in kb, and TPM is the proportion of the gene’s transcripts among per million of total cellular transcripts.

The entire dataset containing 637 beta-cells were randomly divided into 80–20% train-test set. Genes were sorted in descending order according to their expression variability (calculated by ‘median absolute deviation’) in the entire dataset. The top 1000 most variable genes were used for developing a four-layer fully connected neural network (Fig. 1a). The neural network contained two hidden layers with rectified linear unit (ReLU) activation function, and a softmax output layer. The network was trained to classify the pancreatic cells into three chronological stages: ‘Juvenile’ (1 mpf), ‘Adolescent’ (3, 4 and 6 mpf) and ‘Adult’ (10, 12 and 14 mpf). During training, a five-fold cross-validation was repeated three times over a grid of values for regularization hyperparameters: dropout frequency (0.4 to 0.9 in steps of 0.1) and regularization constant (0.4 to 1.6 in steps of 0.2). The combination with the highest cross-validation accuracy was taken as the optimal value, and a final model was trained using the entire training set and the optimal regularization hyperparameters. The entire network was implemented in R using TensorFlow API. An Rmarkdown report detailing the development of zebrafish beta-cell GERAS is available on Github^53^. The trained model was used to classify the chronological stage of the test set. Accuracy was calculated as the proportion of cells for which the classification matched the chronological age. By considering each classification as a binomial distribution (a ‘Juvenile’ cell can be classified as ‘Juvenile’ or ‘Not Juvenile’), the standard error was calculated using the following formula:

$$Standard\,error=\sqrt{\frac{accuracy\ast (1-accuracy)}{n}}$$

where *n* is the number of cells tested.

In addition to accuracy, additional evaluation metrics were also calculated. This included ‘precision’, ‘recall’ (or ‘sensitivity’) and ‘F_1_-Score’. Precision is the ratio of true-positives to the sum of true-positives and false-positives. Recall (or sensitivity) is the ratio of the true-positives to the sum of true-positives and false-negatives. F_1_-Score provides the harmonic mean of precision and recall and equals 2 ∗ (precision ∗ recall)/(precision + recall).

### Classification of chronological stage using GERAS for zebrafish beta-cells

For external validation (1, 4 and 13 mpf and 3 mpf C1-sample) and interpolation (1.5 mpf and 9 mpf), new batches of zebrafish beta-cells were isolated in 96-well plates and sequenced. The one and 13 mpf samples were collected on separate wells of a single 96-well plate and processed together. Quality controlled raw counts were obtained as outlined above. The raw counts were normalized to TPM values, which were then used to classify the chronological stage using pre-trained GERAS. Results were depicted as balloonplots, where a grid contains dots whose size reflects the percentage of cells classified in the corresponding group.

### Assessing the impact of calories on the chronological age of zebrafish beta-cells using GERAS

Twelve zebrafish at 3 mpf from the same clutch were separated into two groups of 6 animals each. Both groups were fed with their normal feed of freshly hatched *Artemia* (brine shrimp). The intermittent feeding group was fed on alternate day, while the other group was fed three times daily with intervals of at least two hours between the feedings. Amount of food eaten by each animal was not controlled. After a month, the beta-cells were isolated into 96-well plates using FACS. The cells were processed and sequenced together. TPM-normalized counts from the cells were used to classify the chronological stage using GERAS.

### Differential gene expression and gene ontology (GO) analysis

Differential gene expression analysis was performed between beta-cells collected from animals fed three-times a day. The beta-cells classified as ‘Adults’ were compared to beta-cell classified as ‘Adolescent’. Differential gene expression analysis was performed ROTS package^43^. For comparison, log_2_-TPM values were used. The cut-off for differential expression was set at false-discovery rate of 0.05. For differentially expressed genes, unbiased gene ontology (GO) analysis was performed using DAVID^42^.

### Statistical analysis

Statistical analysis was performed using R. No animals were excluded from analysis. Blinding was not performed during analysis. Analysis of normal distribution was performed. To compare classification probability between the three stages for 1.5 mpf and 9 mpf samples, one-way ANOVA followed by post-hoc Tukey HSD (Honestly Significant Difference) was performed. To compare chronological age (Adolescent versus Adult) between beta-cell from intermittent feeding and three-times a day fed animals, Fisher’s exact test for count data (fisher.test(x = 2X2 matrix, alternative = “two.sided”)) was performed. To compare the expression levels of *junba* and *fosab* between beta-cells from ‘Juvenile’, ‘Adolescent’ and ‘Adult’ stages, t-test was calculated using the ROTS package^43^. To compare the proliferation between *DN-junba* expressing cells with control cells, an unpaired two-tailed t-test with unequal variance (t.test (x = dataframe, alternative = “two.sided”, paired = FALSE, var.equal = FALSE)) was used to calculate p-values. A p-value of less than 0.05 was considered statistically significant.

### Development of GERAS for human pancreatic cells

For development of GERAS for human pancreatic cells, read counts from Enge *et al*.^46^ were obtained from GEO: GSE81547. Read counts were normalized to reads per million (RPM) using the formula:

$$RP{M}_{gc}=\frac{Read\,Coun{t}_{gc}}{{\sum }^{}Read\,Coun{t}_{c}}\ast 1,\,000,\,000$$

where for gene *g* and cell *c*, RPM_gc_ is the proportion of the gene’s reads among per million of the total cellular reads.

The entire dataset containing 2544 pancreatic cells was randomly divided into 80–20% train-test set. Genes were sorted in descending order according to their expression variability (calculated by ‘median absolute deviation’) in the entire dataset. The top 1000 most variable genes were used for developing a four-layer fully connected neural network (Fig. 4a). The neural network contained two hidden layers with ReLU activation function, and a softmax output layer. The network was trained to classify the pancreatic cells into three chronological ages: ‘Juvenile’ (1 month, 5 and 6 years), ‘Young’ (21 and 22 years), and ‘Middle’ (38, 44 and 54 years). During training, a five-fold cross-validation was repeated three times over a grid of values for regularization hyperparameters: dropout frequency (0.4 to 0.9 in steps of 0.1) and regularization constant (0.2 to 1.2 in steps of 0.2). The combination with the highest cross-validation accuracy was taken as the optimal value, and a final model was trained using the entire training set and the optimal regularization hyperparameters. The entire network was implemented in R using TensorFlow API. An Rmarkdown report detailing the development of human pancreatic GERAS is available on Github^53^. The trained model was used to classify the chronological age of the test set. Accuracy was calculated as the proportion of cells for which the classification matched the chronological age. By considering each classification as a binomial distribution (a ‘Middle’ cell can be classified as ‘Middle’ or ‘Not Middle’), the standard error was calculated using the following formula:

$$Standard\,error=\sqrt{\frac{accuracy\ast (1-accuracy)}{n}}$$

where *n* is the number of cells tested.

To calculate the accuracy and standard error per cell type, the expression levels of the following cell-specific markers were extracted for each cell: ‘INS’ (beta-cell), ‘GCG’ (alpha-cell), ‘SST’ (delta), ‘PRSS1’ (acinar) and ‘KRT19’ (ductal). A cell was classified if the expression value of any cell-specific marker exceeded 50 RPM, else it was classified as ‘Others’. For classification, the cell-type marker with the highest expression determined the cell type. Thus, a (theoretical) cell with RPM values of 1000 INS, 3 GCG, 4 SST, 0 PRSS1, 0 KRT19 was classified as beta-cell, while another (theoretical) cell with RPM values of 3 INS, 5 GCG, 7 SST, 1777 PRSS1, 9 KRT19 was classified as acinar cell. Cell-type specific cells present in the test set were used to calculate the accuracy per cell-type.

F_1_-score was calculated as in the development of zebrafish beta-cell GERAS for all stages and cell-types.

### Independent cohort of human pancreatic cells

For testing GERAS with external data, read counts of pancreatic single-cell data from Segerstolpe *et al*.^47^ were obtained from ArrayExpress (EBI) with accession number: E-MTAB-5061. The publication contained data from six healthy individuals. The entire data was stratified according to the individuals, and cells from each individual that passed quality-control according to Segerstolpe *et al*. were used for further analysis. Read counts from the cells were normalized to RPM for input to GERAS.

### Calculating classification probability for ‘Middle’ (38–54 years) stage

To calculate the probability that a particular cell would be classified to the ‘Middle’ stage, the softmax for the ‘Middle’ stage was calculated from the output layer of human pancreatic GERAS. For this, the function model_softmax is provided with the log2-transformed RPM values and used to calculate the probability for the particular cell to classify in all the three stages (‘Juvenile’, ‘Young’, and ‘Middle’). The probability for ‘Middle’ stage was extracted from this output.

### Classification of chronological stage using GERAS for human pancreatic cells

For classifying the chronological stage of cells belonging to individuals of age 22, 23, 43 and 48 years, RPM values from each individual were used as input to human pancreatic GERAS. Results were depicted as balloonplots, where a grid contains dots whose size reflects the percentage of cells classified in the corresponding group.

## Electronic supplementary material

Supplementary Data

Table S1

Table S2

Table S3

Table S4

Table S5

## Data Availability

The raw datasets, along with tabulated count data and TPM normalized values, generated during the current study are available from GEO under accession number GSE109881. Normalized read-counts for all human pancreatic samples used in the study, and codes for developing and testing GERAS are available on Github^53^. Please refer to README.md to navigate the Github folder. The authors welcome any requests for information on the raw data, data processing, GERAS development and utilization.
