# Supplementary material for: Machine learning based classification of cells into chronological stages using single-cell transcriptomics
Source: Sci Rep. 2018 Nov 21;8:17156. doi: 10.1038/s41598-018-35218-5 (PMC6249247; doi:10.1038/s41598-018-35218-5)
Supplement: Supplementary file 1 — Supplementary Data [file 41598_2018_35218_MOESM1_ESM.pdf]

Supplementary Data

Machine learning based classification of cells into chronological stages using single-cell transcriptomics

Sumeet Pal Singh, Sharan Janjuha, Samata Chaudhuri, Susanne Reinhardt, Annekathrin Kränkel, Sevina Dietz, Anne Eugster, Halil Bilgin, Selçuk Korkmaz, John E. Reid, Gökmen Zararsız, Nikolay Ninov

Includes:

Supplementary Figures S1 – S9

Supplementary Table S1-S5 Legends

Supplementary Methods

Supplementary References

Supplementary Figure S1

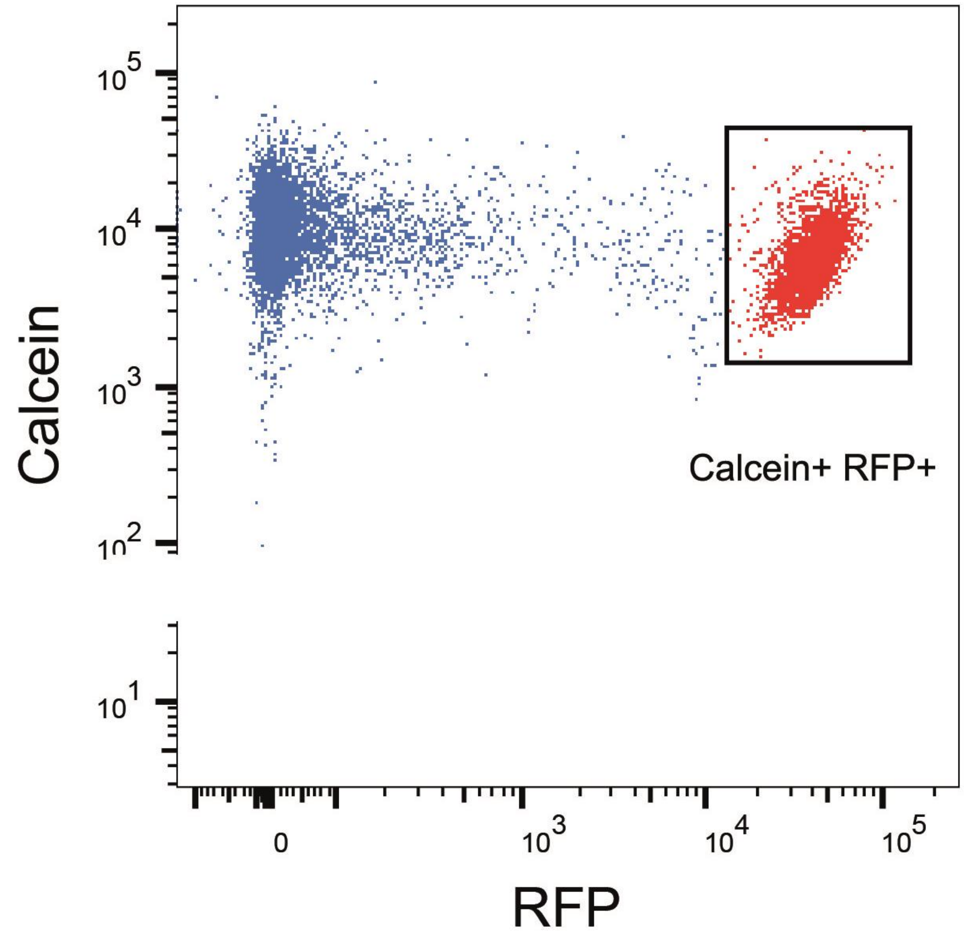

Supplementary Figure S1: FACS sort of zebrafish beta-cells

A FACS plot of live RFP-positive cells from *Tg(ins:BB1.0L)* animals at 3 mpf. Calcein labels live cells, while RFP labels beta-cells.

Supplementary Figure S2

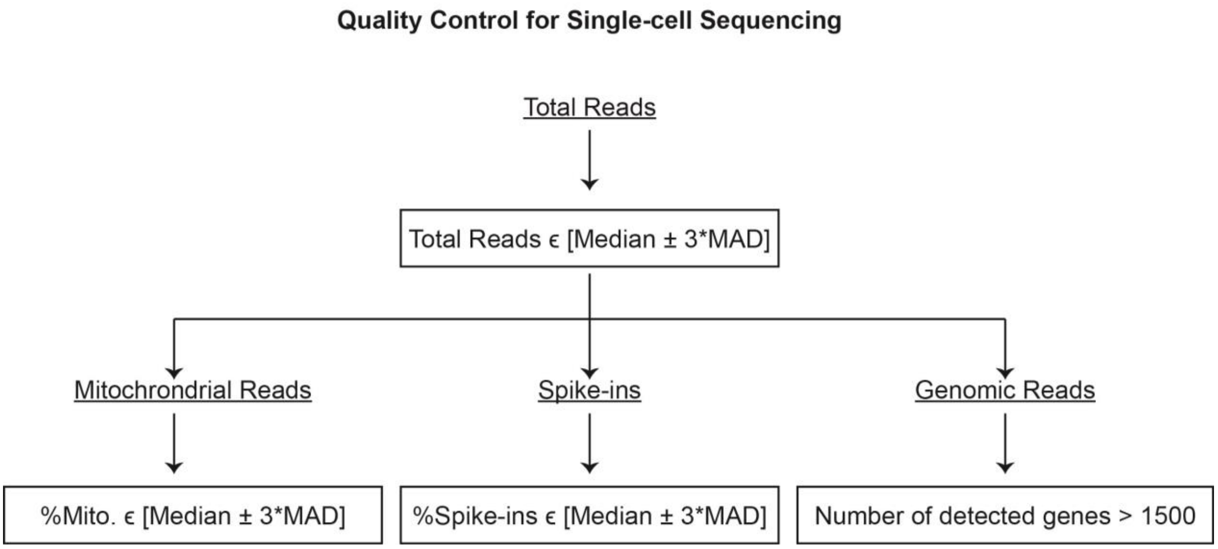

Supplementary Figure S2: Quality control for zebrafish beta-cells used to develop GERAS

A flowchart showing the steps for determining sequencing quality. The following quality control parameters were obtained for the entire dataset:

1. The median and median absolute deviation (MAD) for total reads
2. The median and MAD for % of mitochondrial reads
3. The median and MAD for % spike-ins
4. Number of detectable genes

Cells passed quality control if they belonged to median ± 3*MAD bracket for 1-3 and contained more than 1500 genes.

Supplementary Figure S3

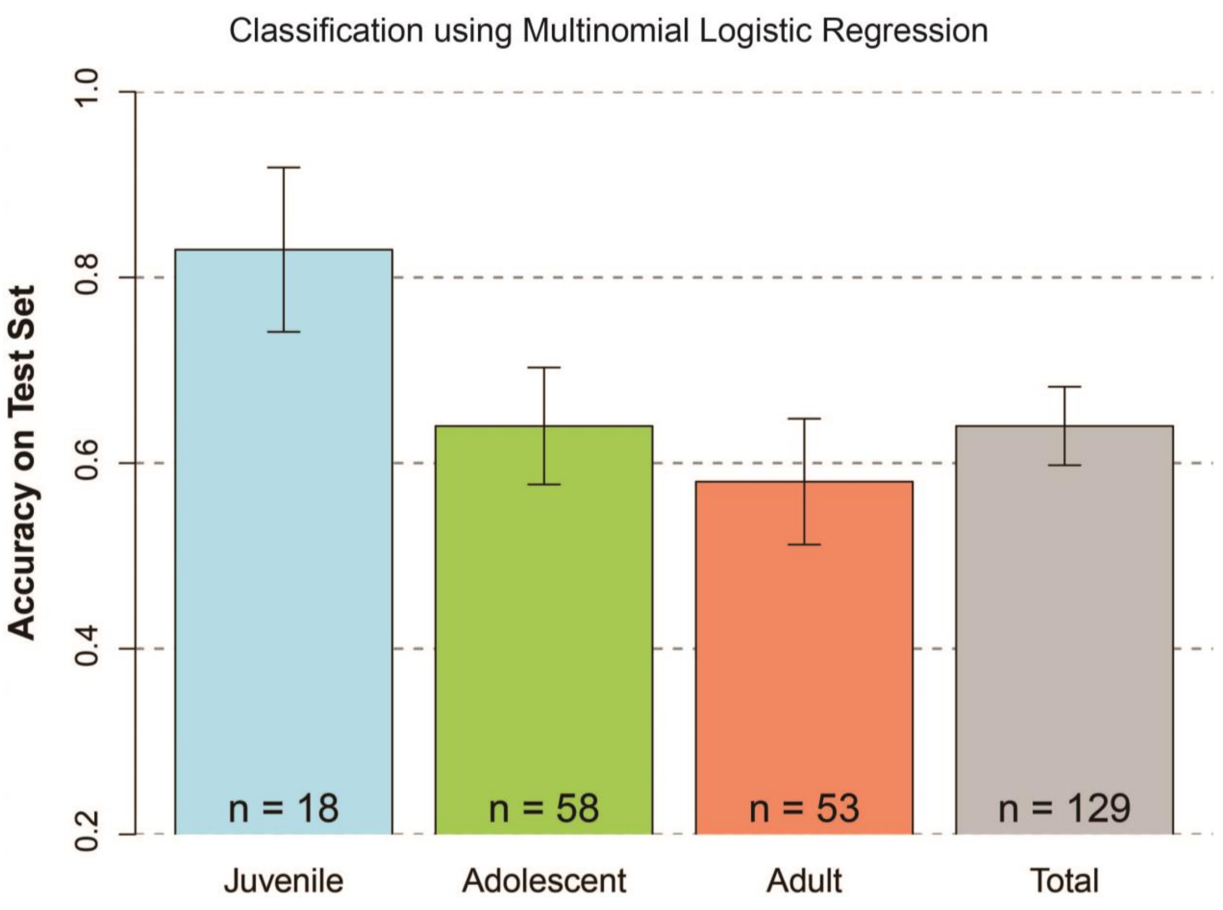

Supplementary Figure S3: Classification of chronological stages using Multinomial Logistic Regression

Barplot showing the accuracy of a Multinomial Logistic Regression for classifying the age of zebrafish beta-cells on the test set – the cells that were excluded from the training of the logistic regression model. The classifications on the test display an overall accuracy of 64%. Error bars indicate standard error. Multinomial Logistic Regression was carried out using the ‘nnet’[1] package in R.

Supplementary Figure S4

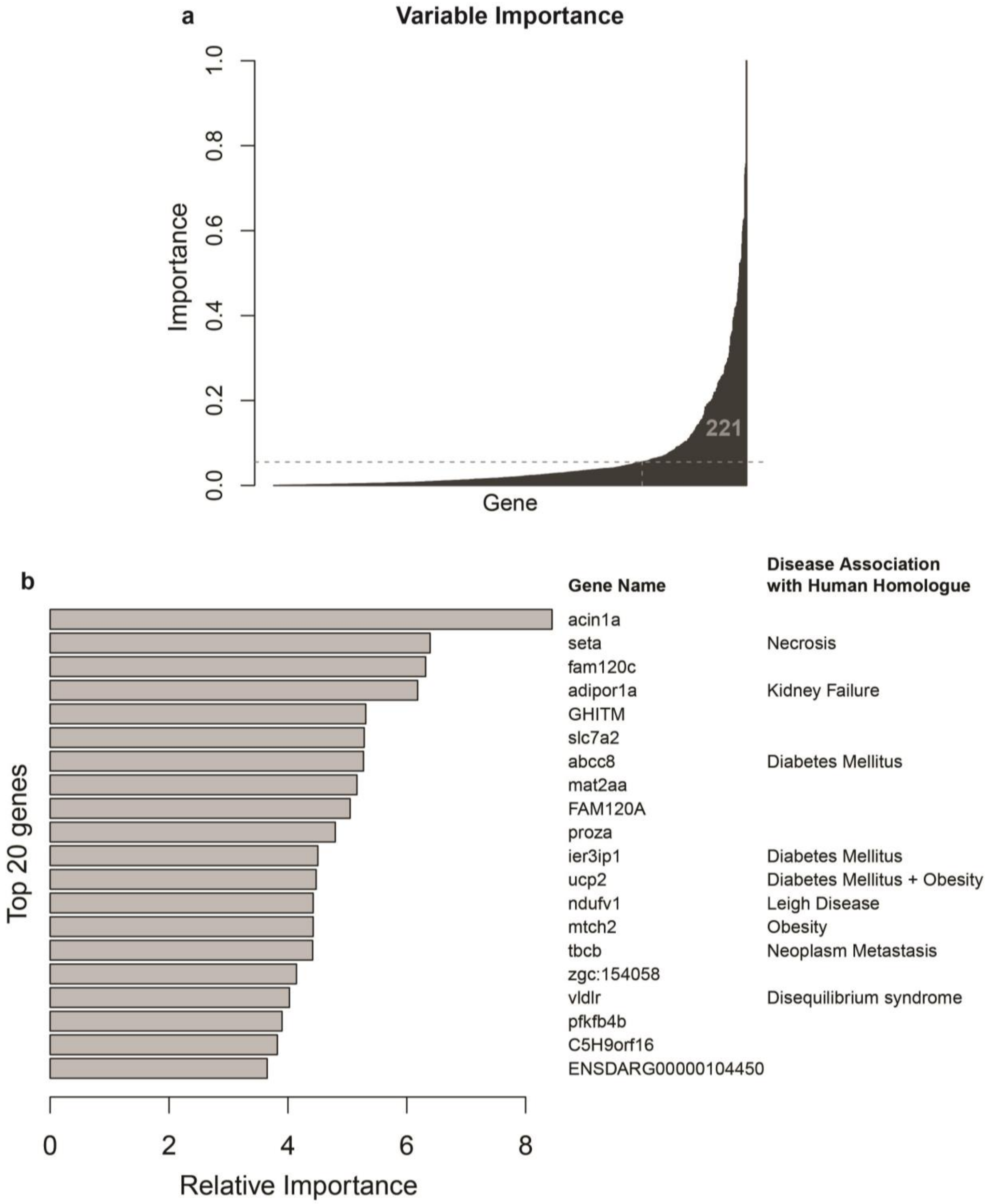

Supplementary Figure S4: Variable importance for zebrafish beta-cell GERAS

(a) Plot depicting the importance of each variable (gene) for the classification using zebrafish beta-cell GERAS. The importance of each input gene is calculated using the strength

(weights) of the neural network connections[2]. An input gene with stronger neural network connections will be more important than another input gene with weaker neural network connections. The Y-axis denotes variable importance, with 1 being most important and 0 being least important. On X-axis, all 1000 genes used as input to GERAS are depicted. Dotted horizontal line depicts the mean of the variable importance. 221 genes (lying right of the vertical dotted line) have importance higher than the mean.

(b) Barplot showing the relative importance of the top 20 genes. The X-axis denotes the relative importance, and the Y-axis lists each gene individually. The names of the genes are listed, along with the disease associated with their human homologue. Diseases association were obtained from DisGeNET database[3].

Supplementary Figure S5

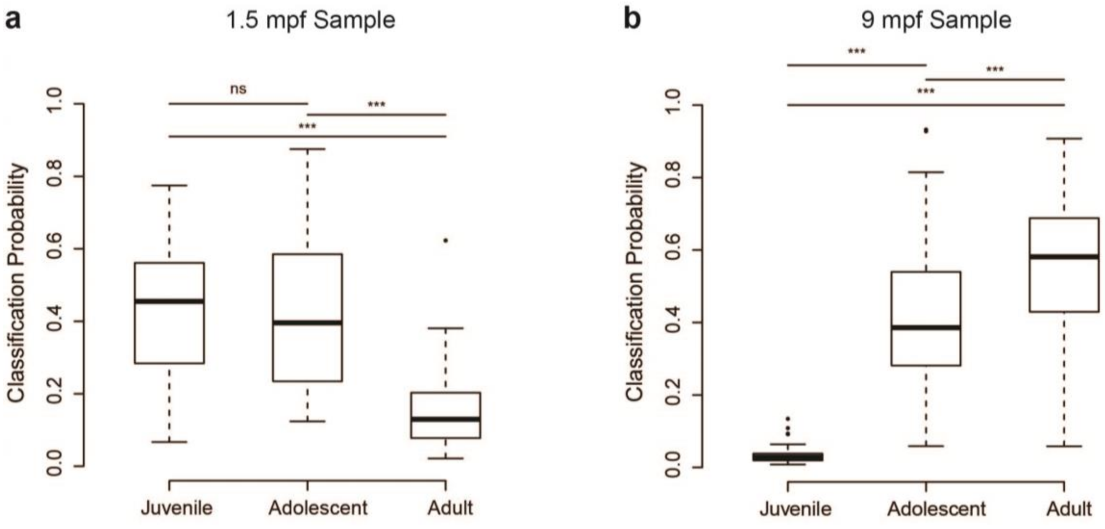

Supplementary Figure S5: Classification probability for the 'Interpolation' samples

(a) Boxplot depicting the classification probability for beta-cells collected from 1.5 mpf animals. The probability for beta-cells from 1.5 mpf animals to be classified into the 'Juvenile' stage (mean = 0.43) is statistically similar to the probability for the samples to be classified into the 'Adolescent' stage (mean = 0.42). In contrast, the samples display a lower probability to be classified into the 'Adult' stage (mean = 0.15). (ANOVA, p-value < 0.001; Tukey's test: ns, p-value > 0.05; *** p-value < 0.001).

(b) Boxplot depicting the classification probability for beta-cells collected from 9 mpf animals. The samples display the highest probability to be classified into the 'Adult' stage (mean = 0.56), followed by classification into the 'Adolescent' stage (mean = 0.41). The samples display the lowest probability to be classified into the 'Juvenile' stage (mean = 0.03). (ANOVA, p-value < 0.001; Tukey's test: *** p-value < 0.001).

Supplementary Figure S6

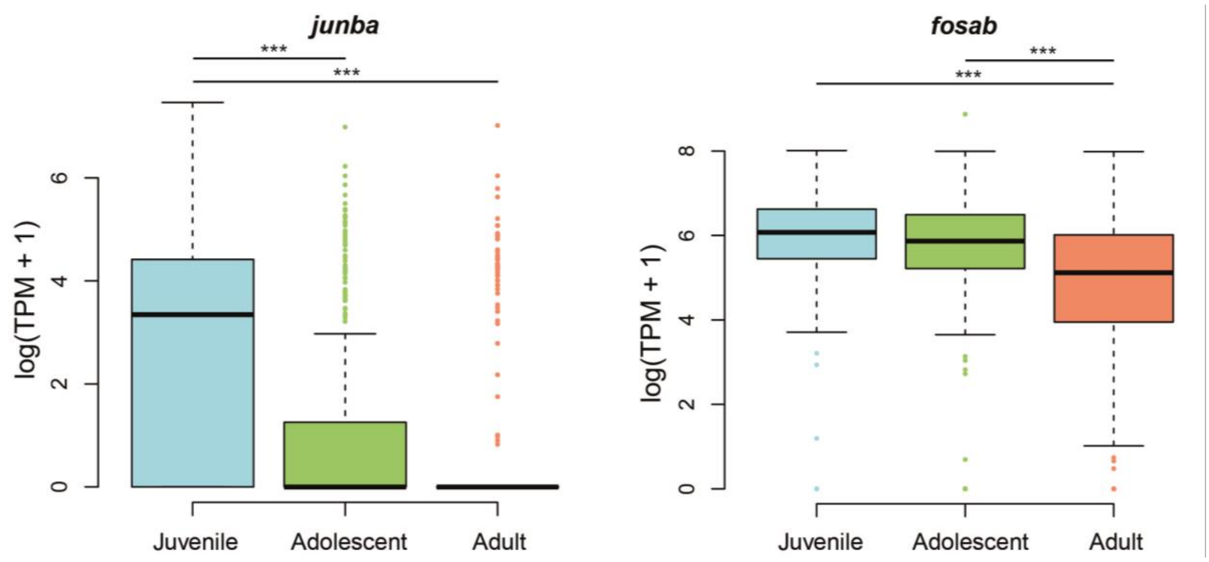

Supplementary Figure S6: Gene expression dynamics with age

Tukey-style boxplots showing expression of *junba* (left) and *fosab* (right) in single beta-cells during aging. Both genes show statistically significant down-regulation with age (t-test using the ROTS package[4], ***p-value < 0.001).

Supplementary Figure S7

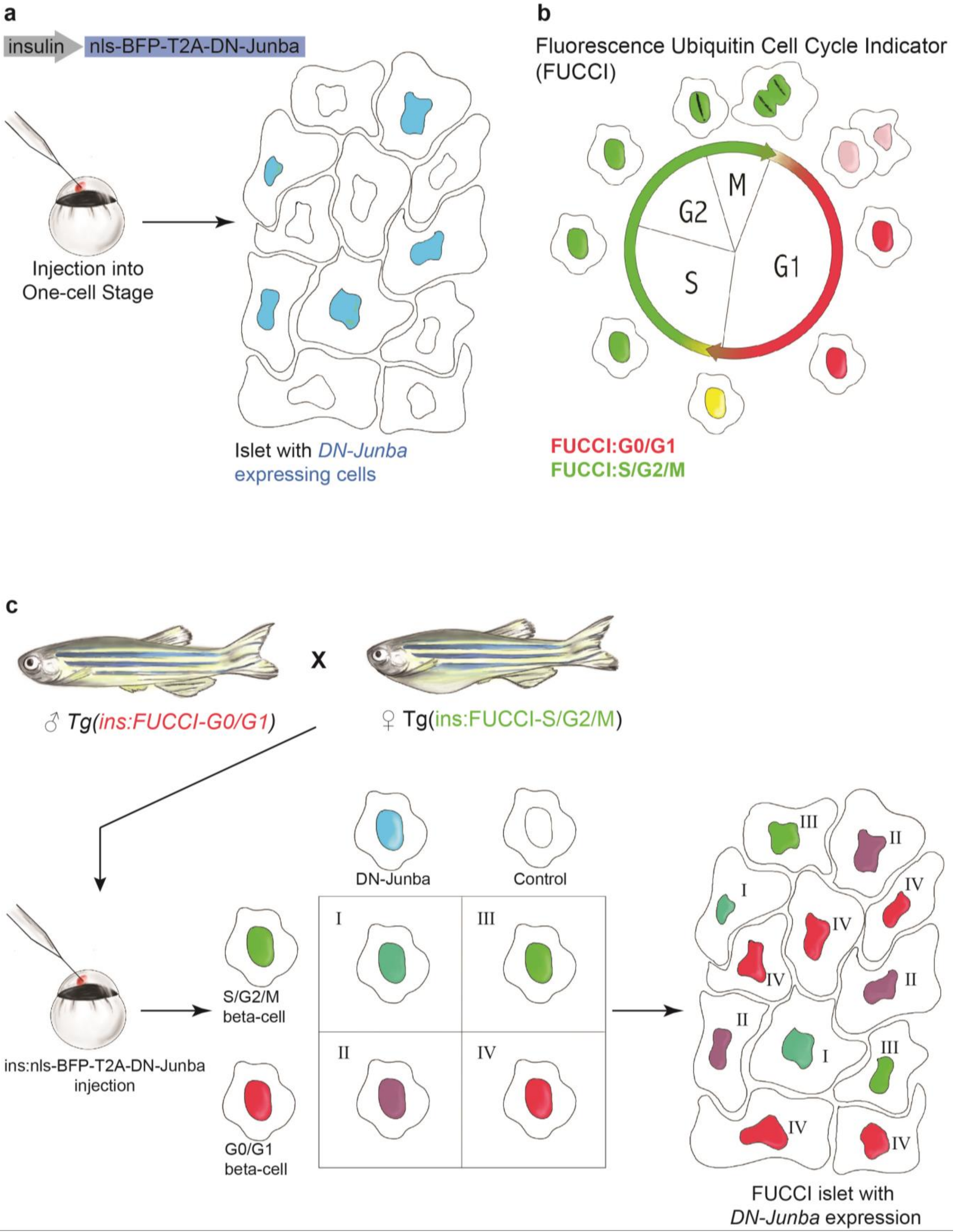

Supplementary Figure S7: Setup of mosaic analysis for evaluating the impact of *DN-junba* on beta-cell proliferation.

(a) Illustration depicting the generation of genetic mosaics. Zebrafish embryos are injected at one-cell stage with a plasmid expressing the blue fluorescence protein tagged *DN-junba* from the insulin promoter. The insulin promoter restricts expression to beta-cells. Random integration of the genetic cassette leads to expression of *DN-junba* in a subset of beta-cells, which are labeled in blue color.

(b) A schematic describing labeling of different cell-cycle states using the fluorescence ubiquitination cell cycle indicator (FUCCI) system. FUCCI system includes two components[5]. The FUCCI-G0/G1 fusion protein is degraded during S/G2/M phase, while FUCCI-S/G2/M is spared. This leads to green fluorescence during S/G2/M phase. In contrast, during G0/G1 phase, FUCCI-S/G2/M is degraded and FUCCI-G0/G1 is spared.

(c) Combining genetic mosaics with FUCCI system allows comparison of proliferation among the *DN-junba*-expressing and control cells within the same islet. In this scenario, injection of plasmid is performed in eggs collected from mating of transgenic animals containing the individual FUCCI components. The injected animals grow to yield islets with *DN-junba*-expressing and non-expressing cells. Proliferation is quantified based on the FUCCI-S/G2/M and FUCCI-G0/G1 reporters.

Supplementary Figure S8

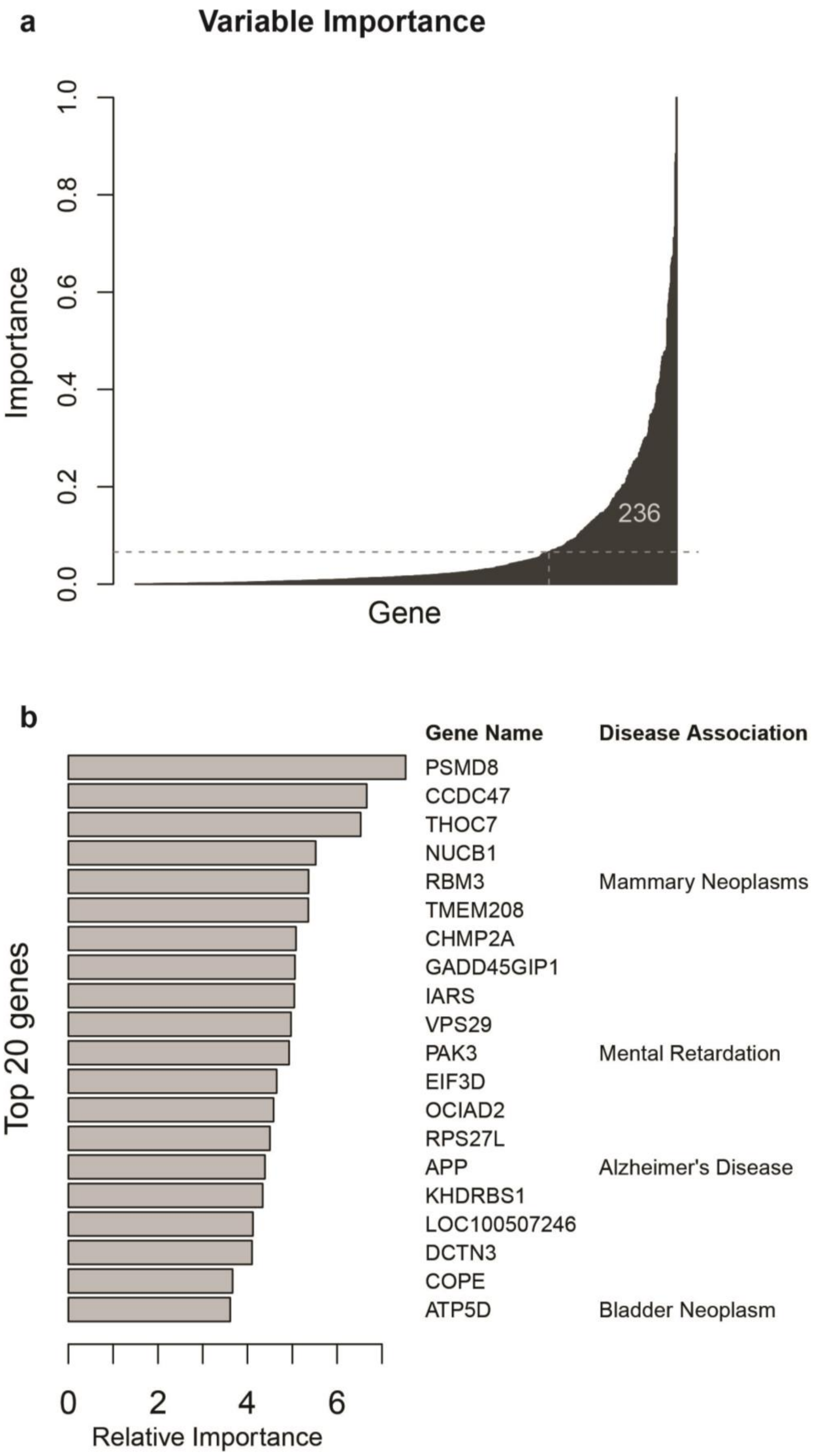

Supplementary Figure S8: Variable importance for human pancreatic GERAS

(a) Plot depicting the importance of each variable (gene) for the classification using human pancreatic GERAS. The Y-axis depicts the importance of the variable from 0 (the lowers) to 1 (the highest). The X-axis shows the 1000 genes used as input to GERAS. The mean of the variable importance is depicted by the dotted horizontal line; with 236 genes (lying right of the vertical dotted line) having higher importance than the mean.

(b) Barplot showing the relative importance of the top 20 genes. Relative importance is plotted on X-axis, and each gene is individually listed on the Y-axis. The names of the genes, along with the disease associated with them is stated on the right. Diseases association were obtained from DisGeNET database[3].

Supplementary Figure S9

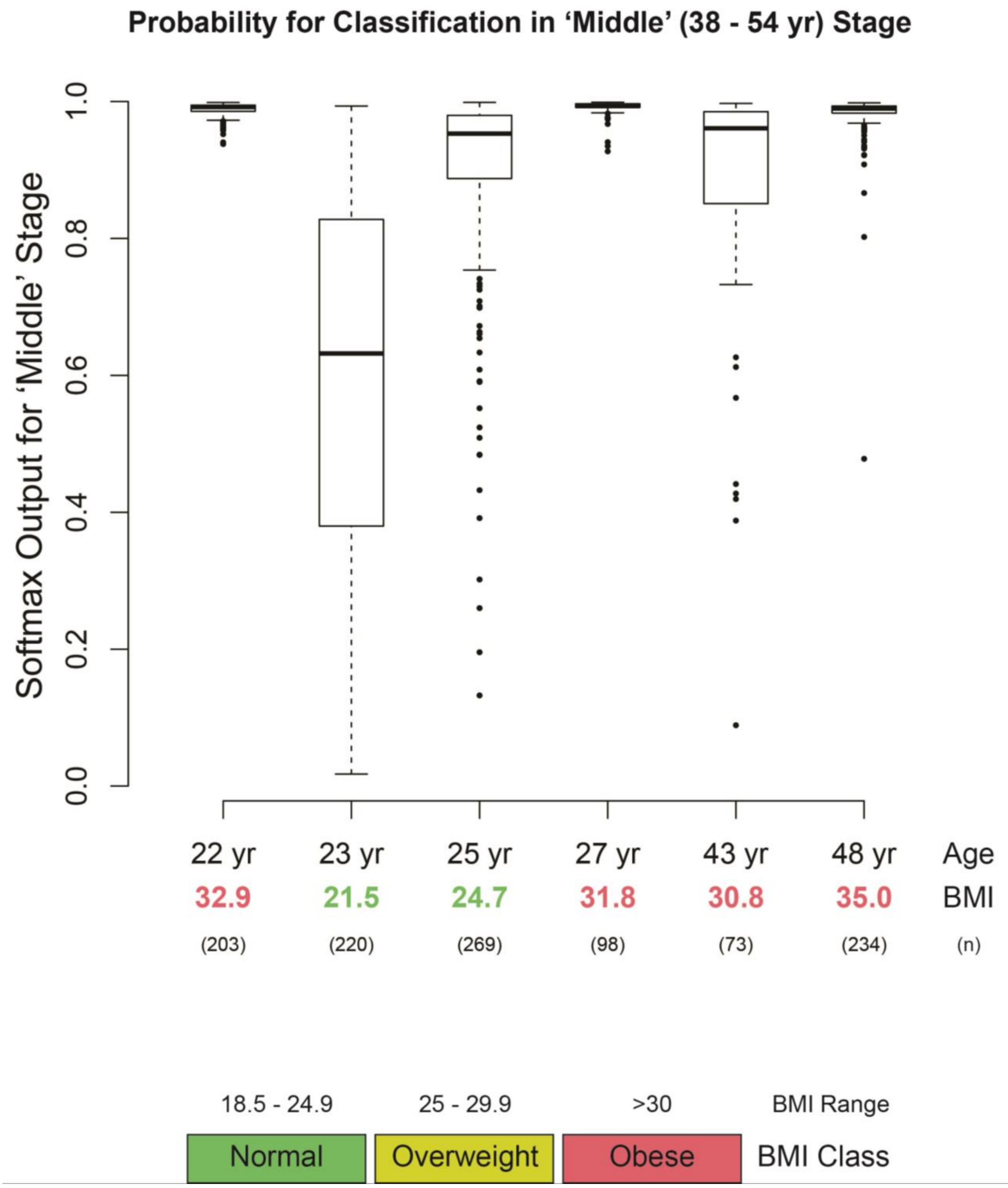

Supplementary Figure S9: Probability of cells from individuals ranging from 22 – 48 years to classify in the ‘Middle’ (38 – 54 years) stage

Tukey-style boxplots showing the softmax output for the data from healthy individuals in Segerstolpe et al.[6] The softmax output for the ‘Middle’ stage of a single cell equals the probability that the cell would classify in the ‘Middle’ stage. Individuals on the X-axis are shown in ascending age. BMI for each individual is shown below the age and color coded according to the BMI ranges indicated below. The BMI ranges are based on world health organization (WHO) recommendations[7]. Numbers of cells from each individual are indicated below the BMI values.

Supplementary Table Legends

Table S1: Samples for development of GERAS for zebrafish beta-cells (.xls)

A table listing the batches in which beta-cells were collected for the development of GERAS for zebrafish beta-cells.

Table S2: Variable Importance for zebrafish beta-cell GERAS (.xls)

A table listing the 1000-input genes utilized by zebrafish beta-cell GERAS and their importance towards classification.

Table S3: Differential gene expression analysis between the beta-cells classified as 'Adult' or 'Adolescent' from zebrafish fed three-times-a-day (.xls)

A table listing the differences in gene expression between beta-cells classified as 'Adult' vs. beta-cells classified as 'Adolescent' from the cells collected from zebrafish fed three-times-a-day. The table lists all the genes in descending order of false-discovery rate (FDR). Genes that show differential gene expression (FDR < 0.05) are italicized.

Table S4: Differential gene expression analysis between the transcriptome of beta-cells collected zebrafish on intermittent feeding vs. the transcriptome of beta-cells collected from zebrafish fed three-times-a-day (.xls)

A table listing the differences in gene expression between beta-cells collected from zebrafish on intermittent feeding vs. three-times-a-day fed zebrafish. The table lists all the genes in descending order of false-discovery rate (FDR). Genes that show differential gene expression (FDR < 0.05) are italicized.

Table S5: Variable Importance for Human pancreatic GERAS (.xls)

A table listing the 1000-input genes utilized by human pancreatic GERAS and their importance towards classification.

Supplementary Methods

Single cell isolation of zebrafish beta-cells

Primary islets from *Tg(ins:BB1.0L; cryaa:RFP)* zebrafish were dissociated into single cells and sorted using FACS-Aria II (BD Bioscience). Islets were dissociated into single cells by incubation in TrypLE (ThermoFisher, 12563029) with 0.1% Pluronic F-68 (ThermoFisher, 24040032) at 37 °C in a benchtop shaker set at 450 rpm for 30 min. Following dissociation, TrypLE was inactivated with 10% FBS, and the cells pelleted by centrifugation at 500g for 10 min at 4 °C. The supernatant was carefully discarded and the pellet re-suspended in 500 uL of HBSS (without Ca, Mg) + 0.1% Pluronic F-68. To remove debris, the solution was passed over a 30 µm cell filter (Miltenyi Biotec, 130-041-407). To remove dead cells, calcein violet (ThermoFisher, C34858) was added at a final concentration of 1 µM and the cell suspension incubated at room temperature for 20 minutes. The single cell preparation was sorted with the appropriate gate for identification of beta-cells (RFP+ and calcein+) (Supplementary Fig. S1). FACS was performed through 100 µm nozzle with index sorting.

Beta-cells were collected from seven ages of zebrafish: 1 mpf, 3 mpf, 4 mpf, 6 mpf, 10 mpf, 12 mpf and 14 mpf. For classification, the seven ages were divided into three chronological stages: ‘Juvenile’ (1 mpf), ‘Adolescent’ (3, 4 and 6 mpf) and ‘Adult’ (10, 12 and 14 mpf). The collection of the beta-cells was carried out in four batches (Supplementary Table S1), with each batch representing a different collection date. In Supplementary Table S1, the four batches (collection dates) are labeled as ‘A’, ‘B’, ‘C’ and ‘D’ for simplification. It is important to note that each batch (except ‘D’) contained samples from more than one chronological stage and conversely each stage (except ‘Juvenile’) was represented in more than one batch.

Single cell mRNA sequencing of zebrafish beta-cells from 96-well plates

Cells were sorted into a 96-well plate containing 2 µl of nuclease free water with 0.2% Triton-X 100 and 4 U murine RNase Inhibitor (NEB), spun down and frozen at -80°C. After thawing the samples, 2 µl of a primer mix was added (5 mM dNTP (Invitrogen), 0.5 µM dT-primer*, 4 U RNase Inhibitor (NEB)). RNA was denatured for 3 minutes at 72°C and the reverse transcription was performed at 42°C for 90 min after filling up to 10 µl with RT buffer mix for a final concentration of 1x superscript II buffer (Invitrogen), 1 M betaine, 5 mM DTT, 6 mM $MgCl2$, 1 µM TSO-primer*, 9 U RNase Inhibitor and 90 U Superscript II. After synthesis, the reverse transcriptase was inactivated at 70°C for 15 min. The cDNA was amplified using Kapa HiFi HotStart Readymix (Peqlab) at a final 1x concentration and 0.1 µM UP primer under following cycling conditions: initial denaturation at 98°C for 3 min, 22 cycles [98°C 20 sec, 67°C 15 sec, 72°C 6 min] and final elongation at 72°C for 5 min. The amplified cDNA was purified using 1x volume of hydrophobic Sera-Mag SpeedBeads (GE Healthcare) and DNA was eluted in 12 µl nuclease free water. The concentration of the samples was measured with a Tecan plate reader Infinite 200 pro in 384 well black flat bottom low volume plates (Corning) using AccuBlue Broad range chemistry (Biotium).

For library preparation, 700 pg cDNA in 2 µl was mixed with 0.5 µl tagmentation enzyme and 2.5 µl Tagment DNA Buffer (Nextera DNA Library Preparation Kit; Illumina) and tagmented at 55°C for 5 min. Subsequently, Illumina indices were added during PCR (72°C 3 min, 98°C 30 sec, 12 cycles [98°C 10 sec, 63°C 20 sec, 72°C 1 min], 72°C 5 min) with 1x concentrated KAPA Hifi HotStart Ready Mix and 0.7 µM dual indexing primers. After PCR, libraries were quantified with AccuBlue Broad range chemistry, equimolarly pooled and purified twice with 1x volume Sera-Mag SpeedBeads. This was followed by Illumina sequencing on a Nextseq500 aiming at an average sequencing depth of 0.5 million reads per cell.

*dT primer: Aminolinker-AAGCAGTGGTATCAACGCAGAGTCGAC T(30) VN

*TSO primer: AAGCAGTGGTATCAACGCAGAGTACATggg

*UP primer: AAGCAGTGGTATCAACGCAGAGT

The C1™ Single-Cell mRNA Seq 10-17 µm IFC (© Fluidigm Corporation, CA, USA) was used to perform mRNA sequencing on single cells. In general, the protocol (PN 100-7168 L1) suggested by the manufacturer was followed, with some modifications. 1200 cells in PBS were directly sorted by FACS into the inlet, mixed 3:2 with suspension reagent, resulting in a final volume of 6 µl. Cells were loaded with the mRNAseq: Cell load protocol, without staining on the IFC. For RT and amplification, the mRNA Seq: RT & Amp script was run with the following cycling parameters: 1x 98ºC 1 min, 5x (95ºC 20-45 sec, 59-49ºC with 0.3ºC increment/cycle 4 min, 68ºC 6 min) 9x (95ºC 20-45 sec, 65-49ºC with 0.3ºC increment/cycle 30 sec, 68ºC 6 min) 7x (95ºC 30-45 sec, 65-49ºC with 0.3ºC increment/cycle 30 sec, 68ºC 7 min) and 72ºC 10 min using SMART-Seq v4 Ultra Low Input RNA Kit for Sequencing (Takara BIO USA, INC.). For library preparation, 2 µl cDNA were mixed with 0.5 µl tagmentation enzyme and 2.5 µl Tagment DNA Buffer (Nextera DNA Library Preparation Kit; Illumina) and tagmented at 55°C for 5 min. Illumina indices were added by PCR with the following cycling conditions: 1x (72°C 3 min, 98°C 30 sec), 12 x (98°C 10 sec, 63°C 20 sec, 72°C 1 min), 1x (72°C 5 min), using KAPA Hifi HotStart Ready Mix and 0.7 µM final dual indexing primers. Libraries were quantified, equimolarly pooled and purified twice with 1x volume Sera-Mag SpeedBeads. Illumina sequencing (75bp SE) was done on a Nextseq500 aiming to achieve an average sequencing depth of 0.5 million reads per cell.

Mapping of read counts and quality control

Raw reads in fastq format were trimmed using trim-galore with default parameters to remove adapter sequences. Trimmed reads were aligned to the zebrafish genome, GRCz10, using HISAT2 [8] with default parameters. htseq-count [9] was used to assign reads to exons thus eventually getting counts per gene. Using cells that were utilized for developing zebrafish GERAS (see next section), the following quality control parameters were obtained (Supplementary Fig. S2):

1. The median and median absolute deviation (MAD) for total reads
2. The median and MAD for % of mitochondrial reads
3. The median and MAD for % spike-ins
4. Number of detectable genes

Cells passed quality control if they belonged to median ± 3*MAD bracket for 1-3 and contained more than 1500 genes. Read counts for all cells that passed quality control are available from GEO under accession number GSE109881.

Development of a Multinomial Logistic Regression Model for zebrafish beta-cells

For development of multinomial logistic regression for zebrafish beta-cells, TPM normalized counts were used from seven ages of zebrafish distributed into three chronological stages: 1 mpf (‘Juvenile’); 3 mpf, 4 mpf and 6 mpf (‘Adolescent’); 10 mpf, 12 mpf and 14 mpf (‘Adult’). The entire dataset containing 508 beta-cells were randomly divided into 80%-20% train-test set. Multinomial Logistic Regression model was developed using the ‘nnet’[1] package in R using the top most variable genes (Supplementary Table S2). With 1000 and 500 top-variable genes, the variance-covariance matrix could not be calculated. Variance-covariance matrix could be calculated for an input of 250 genes. Thus, we developed a model using 250-top variable genes using *model ← multinom (stage ~ gene, data =*

ExpressionData). The code for developing the Multinomial Logistic Regression Model is uploaded as ZF_MultipleLogisticRegression.R on github [10].

The trained model was used to classify the chronological age of the test set. Accuracy was calculated as the proportion of cells for which the classification matched the chronological age. By considering each classification as a binomial distribution (a 'Juvenile' cell can be classified as 'Juvenile' or 'Not Juvenile'), the standard error was calculated using the following formula:

$$Standard\ error = \sqrt{\frac{accuracy * (1 - accuracy)}{n}}$$

where *n* is the number of cells tested.

Construction of the ins:nls-BFP-T2A-DN-junba; cryaa:RFP plasmid

To generate *ins: nls-BFP-T2A-DN-junba;cryaa:RFP*, a vector was created by inserting multiple cloning sites (MCS) downstream of the insulin promoter to yield *ins:MCS; cryaa:RFP*. To do so, the plasmid *ins:mAG-zGeminin;cryaa:RFP* was digested with EcoRI/PacI and ligated with dsDNA generated by annealing two primers harboring the sites EcoRV, NheI, NsiI, SalI and flanked by EcoRI/PacI overhangs. The plasmid pUC-Kan consisting of the DN-junba (junba$^{157\text{-}325}$, consisting of only the DNA binding domain[11]) fused to *nls-BFP* via T2A sequence flanked by EcoRI/PacI sites was synthesized from GenScript. *ins:MCS;cryaa:RFP* and the plasmid *pUC-nls-BFP-T2A-DN-junba* were subsequently digested with EcoRI/PacI to yield compatible fragments, which were ligated together to yield the final construct. The entire construct was flanked with I-SceI sites to facilitate genomic insertion.

Analysis of proliferation using mosaic expression of *DN-junba*

To identify proliferating beta-cells, the zebrafish beta-cell specific FUCCI system[12] was used by crossing *Tg(ins:FUCCI-G1)* with *Tg(ins:FUCCI-S/G2/M)*. Embryos obtained from the mating were injected with *ins:nls-BFP-T2A-DN-junba;cryaa:RFP* plasmid, along with I-SceI, to facilitate mosaic integration into the genome. At 30 dpf, animals were euthanized in Tricaine and dissected to isolate the islets. The isolated islets were fixed in 4% paraformaldehyde (PFA) for 48 hours at 4°C, washed multiple times in PBS and mounted on slides for confocal microscopy. Confocal images were used for cell-counting. All the *Tg(ins*:FUCCI-S/G2/M*)*-positive cells (green fluorescence only) were counted manually within the BFP-positive and BFP-negative clones. Using Imaris (Bitplane), the total number of BFP-positive and beta-cells were calculated in the entire islet. For this, the “spots” function was used after thresholding. For calculating percentages (%), the following calculations were used:

$$Total\,BFP\text{-}negative\ cells = Total\ beta\text{-}cells - Total\ BFP\text{-}positive\ cells$$

$$\%\ BFP\text{-}positive\ proliferating\ cells = \frac{ins{:}FUCCI\text{-}S/G2/M\text{-}positive\ \text{and}\ BFP\text{-}positive\ cells}{Total\ BFP\text{-}positive\ cells} * 100$$

$$\%\ BFP\text{-}negative\ proliferating\ cells = \frac{ins{:}FUCCI\text{-}S/G2/M\text{-}positive\ \text{and}\ BFP\text{-}negative\ cells}{Total\ BFP\text{-}negative\ cells} * 100$$

Calculating variable importance for GERAS

Variable importance was calculated as outlined in Gedeon et al. [2]. The code for carrying out the calculation is shared as source/variableImportance.R on github [10]. The code uses the weights of the trained neural network to calculate the importance of each variable (input) used for classification. The output is scaled to 0 (least important) and 1 (most

important). This was used to identify the importance of each gene used in zebrafish and human GERAS. The results were sorted in descending order for plotting. Additionally, the top 20 most important genes were obtained from the sorted list, and their relative importance calculated using the formula,

$$Relative\ Importance_g = \frac{Importance_g}{\sum_g Importance}$$

where g denotes an individual gene among the top 20. The disease association for each gene was obtained from DisGeNET database [3]. From the database, an association with a score of greater than or equal to 0.2 was reported.

Shiny implementation of GERAS classifier

To enable easy access to classifications using GERAS, a Shiny app was developed. The app is freely available on Github [10]. The app provides a graphic-user interface (GUI) for users to make chronological age classifications using a pre-trained GERAS model. The users can upload normalized counts, verify the uploaded data, and obtain classifications in a downloadable comma-separated (csv) file.

Supplementary References:

1. Venables, W. N. & Ripley, B. D. *Modern Applied Statistics with S*. (Springer, 2002).

2. Gedeon, T. D. Data mining of inputs: analysing magnitude and functional measures. *Int. J. Neural Syst.* **8,** 209–18 (1997).

3. Piñero, J. *et al.* DisGeNET: a comprehensive platform integrating information on human disease-associated genes and variants. *Nucleic Acids Res.* **45,** D833–D839 (2017).

4. Suomi, T., Seyednasrollah, F., Jaakkola, M. K., Faux, T. & Elo, L. L. ROTS: An R package for reproducibility-optimized statistical testing. *PLOS Comput. Biol.* **13,** e1005562 (2017).

5. Sakaue-Sawano, A. *et al.* Visualizing spatiotemporal dynamics of multicellular cell-cycle progression. *Cell* **132,** 487–98 (2008).

6. Segerstolpe, Å. *et al.* Single-Cell Transcriptome Profiling of Human Pancreatic Islets in Health and Type 2 Diabetes. *Cell Metab.* 593–607 (2016). doi:10.1016/j.cmet.2016.08.020

7. World Health Organization. Global database on body mass index. (2011).

8. Kim, D., Langmead, B. & Salzberg, S. L. HISAT: a fast spliced aligner with low memory requirements. *Nat. Methods* **12,** 357–360 (2015).

9. Anders, S., Pyl, P. T. & Huber, W. HTSeq--a Python framework to work with high-throughput sequencing data. *Bioinformatics* **31,** 166–169 (2015).

10. Singh, S. P. GERAS (GEnetic Referene for Age of Single-cell).

https://github.com/sumeetpalsingh/GERAS2017 (2017).

11. Castellazzi, M. *et al.* Overexpression of c-jun, junB, or junD affects cell growth differently. *Proc. Natl. Acad. Sci. U. S. A.* **88,** 8890–4 (1991).

12. Ninov, N. *et al.* Metabolic regulation of cellular plasticity in the pancreas. *Curr. Biol.* **23,** 1242–1250 (2013).
